# Cystathionine Beta-Synthase Deficiency: Three Consecutive Cases Detected in 40 Days by Newborn Screening in Emilia Romagna (Italy) and a Comprehensive Review of the Literature

**DOI:** 10.3390/children10020396

**Published:** 2023-02-17

**Authors:** Egidio Candela, Michele Zagariello, Valeria Di Natale, Rita Ortolano, Francesca Righetti, Valentina Assirelli, Giacomo Biasucci, Alessandra Cassio, Andrea Pession, Federico Baronio

**Affiliations:** 1 Pediatric Unit, IRCCS Azienda Ospedaliero-Universitaria di Bologna, 40138 Bologna, Italy; 2 Specialty School of Pediatrics, Alma Mater Studiorum, University of Bologna, 40126 Bologna, Italy; 3 Centro Laboratoristico Regionale di Riferimento Screening Neonatale e Malattie Endocrino-Metaboliche, UO Pediatria IRCCS Azienda Ospedaliero-Universitaria di Bologna, 40138 Bologna, Italy; 4 The Pediatric Unit, Maternal and Child Department, Guglielmo da Saliceto Hospital, 29121 Piacenza, Italy

**Keywords:** expanded newborn screening, homocysteine, hypermethioninemia, pyridoxine, diet, betaine, cysthiatonine beta-synthase deficiency, LC-MS/MS, classic homocystinuria, CBSD

## Abstract

Cysthiatonine beta-synthase (CBS) deficiency (CBSD) is an autosomal recessive rare disorder caused by variations on *CBS* that leads to impaired conversion of homocysteine (Hcy) to cystathionine. Marked hyperhomocysteinemia is the hallmark of the disease. The administration of pyridoxine, the natural cofactor of CBS, may reduce total plasma Hcy. Patient phenotype is classified on pyridoxine responsivity in two groups: pyridoxine-responsive and non-responsive patients. Ectopia lentis, bone deformities, developmental delay, and thromboembolism are the classic signs and symptoms of the disease. Early diagnosis and treatment impact patients’ natural history. Therapy aims to lower promptly and maintain Hcy concentrations below 100 μmol/L. Depending on the patient’s phenotype, the treatment goals could be obtained by the administration of pyridoxine and/or betaine associated with a methionine-restricted diet. CBSD could be diagnosed in the early days of life by expanded newborn screening (ENS), however, the risk of false negative results is not negligible. In Emilia-Romagna (Italy), during the first 10 years of screening experience, only three cases of CBSD identified have been diagnosed, all in the last two years (incidence 1:118,000 live births). We present the cases and a comprehensive review of the literature to emphasize the role of ENS for early diagnosis of CBSD and its potential pitfalls, reiterating the need for a more effective method to screen for CBSD.

## 1. Introduction

Cystathionine beta-synthase (CBS) deficiency (CBSD), also known as classical homocystinuria (HCU; OMIM 236200), is an autosomal recessive metabolic disorder of sulfur metabolism due to variations on CBS located on chromosome 21q22.3. The deficiency of CBS enzyme activity impairs the conversion of homocysteine (Hcy) to cystathionine. This metabolic derangement leads to an increase in serum Homocysteine (Hcy) and methionine (Met) and reduces the serum levels of cysteine. Pyridoxine is a cofactor of the CBS enzyme that helps the conversion of Hcy to cysteine [1]. There are two forms of CBSD: pyridoxine responsive (PR) and non-pyridoxine responsive (NPR). The genotype–phenotype correlation has been established for some CBS variations and it is based mainly on pyridoxine responsiveness [2]: milder variations would lead to a less severe enzymatic deficiency that would eventually be improved by the administration of pyridoxine.

CBSD usually manifests in the first or second decade of life, however, adult onset is not unusual. The clinical spectrum of untreated CBSD ranges from ectopia lentis, myopia, intellectual disability, osteoporosis, marfanoid habitus, and thromboembolic events [3].

Several publications suggest that patients with CBSD could benefit from early treatment that mainly consists, in responsive cases, of pyridoxine administration in conjunction with betaine and a low protein diet with methionine-free aminoacid supplementations [4,5,6,7]. Therapy aims to rapidly, persistently, and safely reduce total Hcy (tHcy) levels below 100 μmol/L to prevent complications [1]. Therefore, the possibility of an efficient treatment highlights the need for timely diagnoses in a pre-symptomatic phase. This is possible nowadays thanks to the inclusion of CBSD in the diagnostic panels of expanded newborn screening (ENS) programs [8].

The introduction of tandem mass spectrometry (LC-MS/MS) has allowed the evolution of the concept of “newborn screening for a metabolic disease” to that of “expanded newborn screening” [9]. The LC-MS/MS method is a powerful tool in the analysis of amino acids and acylcarnitines from a single drop of blood sample and allows early identification in the neonatal period of more than 100 inherited metabolic disorders, including CBSD [10].

CBSD is generally detected by an increase in Met at ENS, as it is considered the primary marker of the disease, however, this approach is burdened by low sensitivity. The dosage of Hcy is used as a second-tier marker (2TT), evaluated on the same dried blood spot (DBS) in patients with elevated Met at ENS. The main gold standard technique for diagnosis confirmation is the sequencing of the CBS gene [1].

In March 2011, ENS for inborn errors of metabolism using LC-MS/MS has been started in Emilia-Romagna, a region located in central–northern Italy. On 1 January 2022, 4,458,006 people live in Emilia-Romagna [11]. From 2011 to 2021, live births subjected to newborn screening in Emilia-Romagna were 353,253 [12]. All newborns underwent a DBS for ENS at 48–72 h of life at birth centers of the region. The DBS is sent via courier to the regional ENS laboratory in Bologna, where the analysis is performed. Pathological results are sent via a full computerized system to the regional clinical center, which provides to recall the newborns for the diagnostic confirmation procedures.

We report and analyze here in detail the three cases of CBSD identified in our ten years of experience with ENS (2011–2021). Moreover, we review the literature concerning updates in guidelines and worldwide newborn screening CBSD reports. We aim to analyze the potentiality and limitations of ENS in the diagnosis of this pathology.

## 2. Materials and Methods

We reported all the cases of CBSD detected by newborn screening, since Emilia-Romagna included this pathology in the ENS (March 2011).

The case reports were described according to the international CARE guidelines [13]. All patients were identified by detecting elevated Met with a positive second-tier test for hyperhomocysteinemia in DBS (LC-MS/MS chromatography method). The second-tier test, detecting the increase in tHcy, was carried out in our laboratory since 2020. Diagnostic confirmation was performed by mutation analysis of the CBS gene. For incidence calculation, we used data from the annual summary reports about screening (over years 2011–2021) by the Italian Society for the Study of Inherited Metabolic Diseases and Newborn Screening (SIMMESN) [12].

We reviewed the published data about CBSD by performing a comprehensive search through the Pubmed, MEDLINE, and Scopus platforms. These databases were queried using individual keywords and MeSH terms. Keywords were mixed in different combinations using Boolean operators “AND” or “OR” as appropriate and database-related filters to maximize the identification of articles. The entry MeSH terms for the PubMed search were ‘’Cystathionine beta-Synthase Deficiency Disease’’, ‘’Cystathionine beta Synthase Deficiency Disease’’; ‘’Deficiency Disease, Cystathionine beta-Synthase’’; CBS Deficiency; CBS Deficiencies, Deficiencies; CBS, Deficiency and ‘’Homocystinuria’’.

We analyzed guidelines and reviewed articles and case reports. We also checked the references list of relevant studies to identify additional missing studies. Data were extracted by two independent authors (E.C. and M.Z.).

## 3. Literature Review

### 3.1. Epidemiology

The real incidence of CBSD is unknown. The estimated worldwide frequency is 1:344,000, with significant discrepancies in Europe (1:100,000) and in Qatar (1:1800). In the latter case, this probably depends on ethnicity and higher incidence of consanguineous marriages [14].

The results from ENS in Campania, a region in the south of Italy, allowed us to estimate an incidence of 1:77,000 [15].

### 3.2. Pathogenesis

Met is converted to Hcy through three enzymatic reactions, releasing a methyl group used in many substrate methylations. Hcy, which is mainly present in the liver, kidney, brain, heart, and lungs is found in plasma in different chemical forms [16]. There is a protein-bound Hcy, which is about 70% of the total, a free-Hcy (about 1%), and Hcy mixed disulfides with cysteine or other thiols (30%). The set of these three forms is identified as tHcy.

Because of the accumulation of Hcy, this disorder is characterized by lower cysteine blood levels, an increase in remethylation to Met, and higher concentrations of the Met pathway metabolites.

### 3.3. Clinical Presentation

The clinical picture of CBSD could be characterized by ocular, skeletal, vascular, and central nervous systems involvement. However, patients’ phenotypes could range from severe forms, where all four systems are affected, to less severe or even asymptomatic cases [17]. Clinical phenotypes at diagnosis probably depend on the severity of CBSD and the age of the patient. There is scarce data on patients who are very early diagnosed with CBSD and not by ENS. In the reported cases, diagnosis was made by the clinical suspect, incidentally, or by family-targeted screening [18].

The two phenotypic variants of class”c CB’D are classified as B_6_-pyridoxine-responsive and B_6_-pyridoxine non-responsive and have different natural histories and management.

#### 3.3.1. Pyridoxine-Responsive

Pyridoxine-responsive CBSD is typically less severe than non-responsive one. The ocular system involvement is mainly characterized by myopia and ectopia lentis. In most untreated patients ectopia lentis occurs by age of eight years. Ectopia lentis usually occurs earlier in B_6_ non-responsive patients [19,20]. Regarding the skeletal system, patients usually are slender and tall, with a marfanoid habitus. They can also have arachnodactyly, scoliosis, and dolichostenomelia, and they are usually prone to osteoporosis, especially of the vertebrae and long bones; about 50% of affected children show signs of osteoporosis by their teens [21]. The leading cause of early death is thromboembolism. Elevated Hcy levels lead to thrombosis via numerous mechanisms such as reduced anticoagulant processes, increased tissue factor expression, increased thrombin generation, increased platelet reactivity, impaired fibrinolytic potential, and direct vascular injury, including endothelial dysfunction [16].

Cerebrovascular accidents have been described in infants, but they typically affect adults. Venous thrombosis is more common than arterial, and cerebral venous sinus thrombosis has been reported as a presenting sign in childhood [22]. Thromboembolism can manifest also as pulmonary embolism, stroke, and myocardial infarction. The risk of having a vascular event is 25% before 16 years of age and 50% by 30 years of age [23]. Between PR individuals, a vascular event can be the presenting feature of the pathology [24].

CBSD can lead to intellectual disability, with a wide IQ range that ranges from 10 to 138. Pyridoxine-responsive individuals are more cognitively intact or only mildly affected [25]. Seizures occur in 21% of untreated individuals. Some patients can also have behavioural problems, such as personality disorders, depression, anxiety, and psychotic episodes (which can be a presenting sign in adolescence) [26,27]. Harker et al. reported reduced survival and an abnormally rapid turnover of platelets, fibrinogen, and plasminogen [28].

#### 3.3.2. Pyridoxine Non-Responsive

The pyridoxine non-responsive form is the most severe phenotypic variant of CBSD, with many of the symptoms of the disease starting earlier than the B_6_ responsive form. Ectopia lentis usually occurs earlier in PNR patients [20]. Untreated or PNR patients usually have learning difficulties. Extrapyramidal signs, such as dystonia, may affect these patients [26]. Cochran et al. (1990) described an unusual presentation of the PNR variant: a boy of normal intelligence had had asthma from infancy with frequent exacerbations and hospitalizations since 8 years of age, and he was hospitalized at 14 for recurrent left pneumothoraces requiring chest drains. Soon after, he developed a right pneumothorax and, subsequently, a superior sagittal sinus thrombosis with papilledema and right hemiparesis as well as deep venous thromboses [29]. Spontaneous pneumothorax had been reported in two other patients with a PNR form [30]. Other clinical gastrointestinal features, including acute pancreatitis and chronic diarrhoea, were found in patients with the PNR variant [31]. CBS is the first enzyme in the glutathione (GSH) synthesis pathway, and GSH is the principal intracellular antioxidant, which acts by scavenging reactive oxygen. Therefore, the depletion of GSH impairs the redox balance, leading to a large accumulation of reactive oxygen species (ROS) in cells. Yuan and Wei demonstrated that pancreatic damage is improved by vitamin B12, which stimulates the clearance of ROS by conserving GSH [32].

Levy et al. (2002) reported the results of 15 pregnancies in 11 women with CBSD, 6 of whom were PR and 5 PNR. Although the sample size is small, no relationship between the severity of the two variants to either the pregnancy complications or the offspring outcomes was found [33].

### 3.4. Diagnosis

The diagnostic hallmark of CBSD is represented by increased levels of plasma tHcy (>100 μmol/L), associated with a slight increase in Met and decreased levels of cysteine and cystathionine [1].

Free Hcy (fHcy—not bounded homocysteine), which has been possible to measure since the 1960s, is a marker with low diagnostic sensitivity because it can be detectable only when tHcy is over 50–60 μmol/L. Disulfide-Hcy urinary levels are not sensitive enough, because it becomes detectable only when tHcy is over 150 μmol/L [34]. In untreated patients, tHcy is usually over 100 μmol/L, but patients with milder variants of CBSD can present with lower levels. It should be emphasized that tHcy levels can considerably vary depending on pyridoxine levels in PR forms. Most individuals who are heterozygous for CBS variants can have normal fasting tHcy, but their urinary tHcy concentrations may be increased. To support diagnosis, it is possible to measure plasma levels of Met together with folate and Vitamin B12.

It is important to exclude the intake of pyridoxine before biochemical testing because it decreases tHcy plasma levels. Fortified foods and drinks containing pyridoxine should be avoided for 1–2 months (or for at least 2 weeks) before plasma sampling [35]. Sample separation and storage can also influence biochemical testing [36]. Fed state can compromise the diagnosis of CBSD: a small meal will not influence tHcy concentrations in healthy people, but, instead, a protein-rich meal may increase plasma tHcy concentration by 15% after 6–8 h. Moreover, there is a diurnal variation, with the lowest concentrations in the first part of the morning and the highest in the evening. Plasma tHcy probably does not have a seasonal variation. [37,38].

In some patients, the diagnosis of CBSD deficiency could be particularly challenging because serum tHcy concentrations can be normal. To overcome false negative results in highly suspicious CBSD cases, it is possible to determine the cystathionine production in cultured fibroblasts using radioactive substrates. However, sometimes, the activity of CBS in fibroblasts may present as normal in patients with mild enzymatic defects. CBS gene molecular analysis could contribute to diagnosis confirmation bearing in mind that unfortunately up to 10% of pathogenic variants may not be detected [39].

Prenatal diagnosis can be possible by testing CBS activity in cultured amniocytes but not in chorionic villi [40].

Over 160 pathogenic CBS variants have been found, many of them typically recurrent in some specific populations such as c.1006 C > T (p.R336C) in Qatar, where it is associated with a serious pyridoxine non-responsive form of CBSD. The most widespread variation c.833 T > C (p.I278T) codifies a mild pyridoxine-responsive enzymatic deficiency in the homozygous state [18].

### 3.5. Expanded Newborn Screening

Elevated Met is the primary marker of the disorder, but we can also detect hyperhomocysteinemia and an increased Met-to-Phe ratio in DBS. Total homocysteine has rarely been used as a primary marker.

For pyridoxine non-responsive forms, the sensitivity of Met as the primary marker depends on the cut-off chosen. For pyridoxine-responsive forms, the sensitivity of that marker is probably very low. The specificity of Met can be improved by adding tHcy as a 2TT and calculating the Met/tHcy ratio [1].

Despite good results in identifying CBSD by ENS classically, based on methionine levels, false negative results have been reported. This is probably due to the low sensitivity of the cut-offs used. Moreover, the false negative results could also depend on the severity of CBS enzymatic activity, as the exact proportion of cases missed by NBS for Met is unknown but considered up to 20–50% of those with pyridoxine non-responsive with DBS obtained during the 36th and 72nd h of life [14]. The laboratory data at the time of diagnosis in 328 patients with CBS deficiency from the E-HOD (European network and registry for Homocystinurias and methylation Defects) registry showed that plasma tHcy and free homocysteine did not differ significantly among the responsiveness groups while, in contrast, plasma or serum Met decreased significantly with increasing responsiveness [41]. By reducing methionine cut-off level, the number of false negatives would decrease, but, in turn, the false positive rate would increase. Several ENS programs have recently reduced the Met cut-off level to 45 µmol/L, just beyond the normal neonatal Met level of nearly 34 µmol/L [42].

The most effective method to reduce the false negative results without increasing the false positive rates probably consists of reducing the cut-offs for the methionine or methionine/phenylalanine (Met/Phe) ratio or both adding a 2TT with tHcy, which requires, however, more effort and the cost of additional LC-MS/MS analyses [42]. In Qatar, where the incidence of that disorder is higher than in other parts of the world, tHcy is utilized as a first-tier test [14].

Hyperhomocysteinemia and Hypermethioninemia, however, could not be associated with CBSD only. Other diseases and nutritional conditions or certain drugs lead to elevated tHcy serum levels. The most common nutritional causes of hyperhomocysteinemia associated with low Met are vitamin B_12_ and folate deficiencies. These conditions can be excluded by measuring serum vitamin B_12_, folates, transcobalamin, and plasma or urinary organic acid (such as methylmalonic acid). tHcy is also elevated in folate deficiency, especially in patients with the MTHFR gene homozygous variant c.667 C > T. Hypermethioninemia can be caused by variations in the GNMT, AHCY gene, or in the main liver metabolizer methionine adenosyltransferase (MAT), which has three subtypes: MAT I, MAT II, and MAT III.

These genes codify for enzymes involved in the different steps of methionine breakdown [43,44,45,46]. In a 2017 article, Maase et al. presented a new LC-MS/MS method for the detection of DBS, with improved specificity and reduced false positive rates [47].

In particular, the *GNMT* gene provides instructions for building the enzyme glycine N-methyltransferase. This enzyme starts to convert AdoMet to a compound called S-adenosyl homocysteine (also known as AdoHcy). On the other hand, the *AHCY* gene codifies for the enzyme S-adenosylhomocysteine hydrolase, which converts AdoHcy into Hcy. Homocysteine may be converted back to methionine or into cysteine, another amino acid [45].

### 3.6. Treatment

The goal of the therapy for early diagnosed patients is to prevent all the complications of the disorder, leading to normal growth and cognitive development.

The main objective of treatment is to reduce plasma tHcy to safe levels and reach normal concentrations of Met and other amino acids. Establishing pyridoxine responsiveness is the first standard procedure in newly diagnosed patients.

The patients are defined as “pyridoxine-responsive” if standard pyridoxine doses can reduce plasma tHcy levels below 50 μmol/L, partial responders if plasma tHcy could be reduced below 50 μmol/L only if a low Met diet is associated with pyridoxine, and “pyridoxine non-responders” when tHcy cannot be reduced either by pyridoxine or a standard low Met diet. The latter cases are more difficult to manage since a severe Met restriction diet can lead to impaired cognitive development and growth failure, while poor metabolic control increases the risk of early thromboembolic events or other complications, as reported by previous Irish studies [48]. The 2017 guidelines group suggests that keeping tHcy under 100 μmol/L is sufficient to prevent complications, which is often a target difficult to reach without compromising the nutritional status [1]. It is important to underline that the target level should be lower (below 60–70 μmol/L) if DBS is used for follow-up [1].

Before starting pyridoxine treatment, tHcy baseline plasma concentrations should be measured twice; then, plasma levels should be measured 1 and 2 weeks after the start of treatment. A blood sample should be taken when a patient is not in a catabolic state and without restricted protein intake. Vitamin B12 and folate plasma levels are often reduced in patients with CBSD deficiency, probably due to the increased activity of the remethylation pathway. Thus, tHcy should be tested after correcting vitamin B12 and folate potential deficiency [1,49].

Treatment starts with 10 mg/kg/day of pyridoxine (500 mg/day maximum) for 6 weeks. If tHcy plasma concentrations after two weeks of treatment reduce below 50 μmol/L in both samples, the patient is considered to be fully pyridoxine responsive, and no other treatment is required.

If tHcy reduces more than 20% from pre-treatment levels but it is still above 50 μmol/L, the patient is partial responder, and a Met diet restriction and/or betaine should be started. If tHcy falls < 20% after 2 weeks of therapy, the patient is considered pyridoxine unresponsive [1]. There is no evidence that long-term therapy with pyridoxine is beneficial in unresponsive patients who do not reach a biochemical response in the first two weeks of therapy. In recent years, a new form of pyridoxine responsiveness has been recognized: some patients are defined as “extreme pyridoxine responders” when they require only a small daily dose of pyridoxine (below ≈ 1 mg/kg/day) to lower plasma tHcy concentrations below 50 μmol/L [41,50].

Moreover, in newborns detected by ENS, it has been recommended by recent guidelines [1] to evaluate pyridoxine responsiveness starting with higher pyridoxine doses of 100 mg/day for 2 weeks, because they rarely respond to the standard dose of 10 mg/kg/day.

The most important adverse effect of pyridoxine is represented by peripheral neuropathy, occurring more frequently in patients long-time treated with a high dose of pyridoxine (above 900 mg/die). Neuropathy has not yet been reported for pyridoxine doses under 500 mg/day [51]. Apnoea and rhabdomyolysis have been also reported as severe adverse effects [52,53].

Dietary management can be used as adjunctive therapy along with betaine and/or pyridoxine. In unresponsive patients, biochemical targets can only be achieved with a diet with low natural protein and with supplements of a Met-free L-AA mixture. Betaine treatment can help patients who do not manage to adhere to dietary treatment because it reduces tHcy levels, allowing an increase in Met diet intake. The most common treatment for unresponsive patients is a combination of diet and betaine [54]. Studies have shown that loss of metabolic control brings serious complications, thus diet treatment must continue throughout life. However, compliance with Met restriction or betaine supplementation often deteriorates, especially in adolescence, or is poor in individuals diagnosed later in life [55].

Betaine is a methyl-derived amino acid compound, and it acts as a methyl donor in the remethylation pathway. It is a nutrient contained in a variety of foods but can also be synthesized in liver and kidneys mitochondria from its precursor choline [56]. In CBSD, betaine is recommended as an adjunctive treatment when diet treatment and pyridoxine are not able to lower Hcy plasma levels below therapeutic recommendations. Betaine treatment significantly reduces tHcy levels and prevented vascular and other complications. In addition to converting tHcy to Met, betaine may also act as a chemical chaperone and correct partial misfolding of CBS mutants [57,58]. Because it increases Met levels, it cannot be used alone to achieve target tHcy concentrations. It seems that patients with Met levels > 80 μmol/L respond less well to betaine therapy [59]. Thus, it should be used as an additional treatment in pyridoxine partially responsive patients who do not reach good control with dietary treatment.

## 4. Results

### 4.1. Our Experience

The three diagnoses of CBSD were all made in about 40 days in the period between April and May 2021. Therefore, the incidence of pathology in Emilia-Romagna over the ten years (2011–2021) of ENS is 1 per 118,000 live births [12]. All patients were term infants, born after an uneventful pregnancy, with a normal adaptation to extrauterine life. The patient’s parents agreed to participate in ENS, which included the measurement of methionine at 48 h of life by DBS, according to national guidelines. They were routinely discharged from the hospital around 72 h postpartum. Patients showed elevated Met levels at the first-tier test, which was confirmed by positive Hcy at 2TT. Therefore, they were recalled at the clinical regional center for diagnosis confirmation by dosing plasma tHcy and Met. The Met cut-off of our laboratory on DBS in Emilia-Romagna was 45 micromol/L while pathological Hcy was over 5 μmol/L. In Table 1, we reported the laboratory values of the first DBS (MET and Hcy) and the blood sampling performed at the diagnostic confirmation.

One patient has consanguineous parents. Sequencing of CBS revealed one homozygous pathogenic variant and 4 other heterozygous variants (shown in Table 2).

A trial of high-dose pyridoxine (100 mg/day) was performed in all patients for 14 days. Two patients (patients 2 and 3) were one partial responder and one non-responder. Patient 1 had a minimal response (−12.6%) at the end of the trial, therefore, although they could not be defined as a partial responder, we maintained the pyridoxine therapy, postponing the decision on its withdrawal. The tHcy values following the trial are shown in Table 3.

Because of the persistence of the Met values above 50 micromol/L, we started a diet therapy with complete Met washout after the trial and progressive Met reintroduction in all patients. The current therapy is the same for all while maintaining a different dietary restriction of Met (Table 3). We measured amino acids and visited the patients approximately every month in the first year of life, then every 3 months. A total of 1/3 of patients experienced problems in feeding due to low palatability of the amino acid supplementation. An attempt was made to shift with other medical foods but without success, thus they have now resumed the initial supplement.

**Figure 1:**
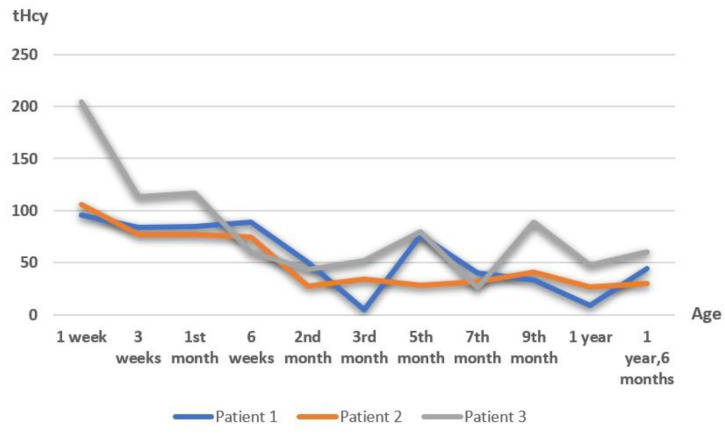
The trend in p-homocysteine. The patient’s age is indicated on the abscissa. Total homocysteine (normal value 5–15 μmol/L) is shown in the ordinate.

The dose of betaine was adjusted according to the tHcy and Met levels. The trend in plasma tHcy (p-homocysteine) levels is schematized in Figure 1.

The three patients are now undergoing a neurological and ophthalmological clinical follow-up, and, at present, they show no signs of disease with satisfactory metabolic control.

### 4.2. Proposal Flow-Chart

**Figure 2:**
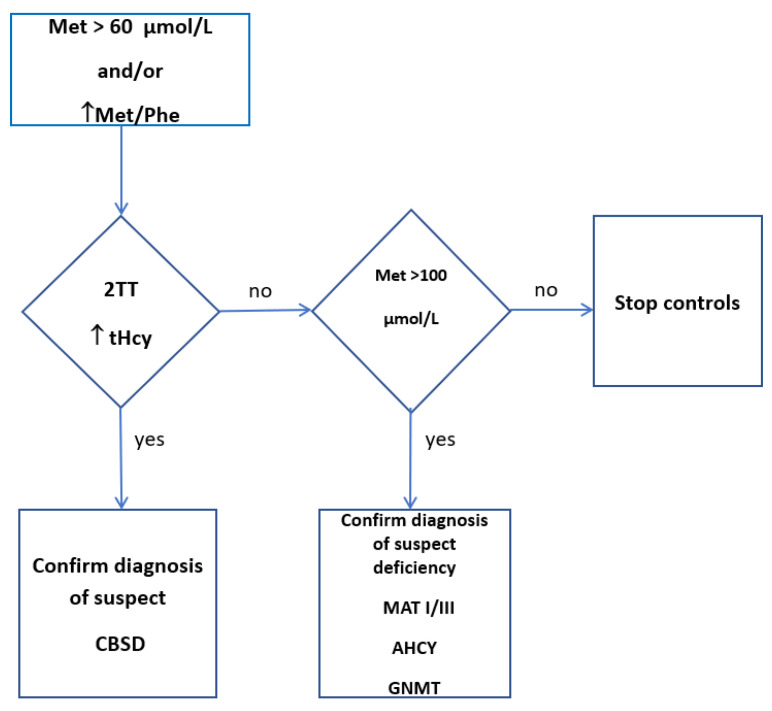
Proposal for a screening flow-chart in patients with hypermethioninemia. Met: Methionine. Met/Phe: methionine/phenylalanine. 2TT: second-tier test. Hcy: Homocysteine. MAT I/III: Methionine adenosyltransferase I/III. AHCY: S-adenosylhomocysteine hydrolase. GNMT: glycine N-methyltransferase. CBSD: Cystathione Beta-Synthase Deficiency.

Our proposal for a screening flowchart is made in Figure 2.

## 5. Discussion

CBSD has been surprisingly diagnosed by ENS in three patients in Emilia-Romagna over a very short period of observation (a couple of months in 2021), whereas no diagnosis has been made in the previous 10 years. Interestingly, according to the technical report of SIMMESN in the years 2020–2021, no other diagnosis of CBSD by ENS has been made in Italy [12]. Therefore, although limited, this case series is truly remarkable.

Classical homocystinuria is a well-known multisystem disease that all clinicians should be aware of. This pathology appears to be a significant prototype of severe pathology that can be detected by ENS with important benefits [4,5,6,7]. In Italy, between 2016 and 2017 with Bill 167, the government decided to extend by law the number of inherited metabolic disorders that all newborns, all over the country, undergo free of charge to harmonize regional differences [63]. Nowadays, in the official panel, there are 47 inherited metabolic disorders. In Emilia-Romagna, one of the wealthiest and most developed regions in Europe [64] since March 2011, the metabolic panel was expanded with a pilot project to all these new pathologies. We are unable to find any convincing explanation for such a diagnostic exploit except for the implementation of the 2TT with Hcy from the year 2020 in our center. We cannot exclude a priori that the availability of an immediate 2TT could have increased the number of diagnoses in our region among patients with elevated Met at ENS. Unfortunately, we have not yet performed a re-check of dried blood spots for the tHcy of those cases with elevated Met that have been classified as not affected by CBSD in the previous years.

The incidence of CBSD in Emilia-Romagna (1 per 118,000 live births) is not far from the data of the Campania region, which, at the time of publication of the data, screened on the basis of hypermethioninemia alone without 2TT [15].

We cannot evaluate the specific benefits of CBSD newborn screening on our patients yet. An article from 1996 by Cruysberg et al. showed how the patients diagnosed by family targeted screening, followed up with and treated from birth to late childhood and adulthood, did not present any complications [65]. Our patients are all, currently, asymptomatic with normal psychomotor development. The incidence of complications, which we know to be characteristic of the second decade of life [20,21,22,23], cannot yet be evaluated as the ENS has only been active for this pathology for 10 years and our only three patients, specifically, are less than 2 years old. Therefore, only a longer follow-up will allow us to evaluate the impact of ENS on the outcome of these patients. Data from the 2011 survey in 16 European countries and other studies cannot be easily compared to our series due to the different grouping of disorders and because they currently focus more on the ability to diagnose the pathology than on a long clinical follow-up [66]. For the same reason, we are unable to carry out a complete genotype–phenotype correlation, in our series, except for biochemical aspects, such as the response to pyridoxine. Patient 3, carrier of the two new variants c.1333 C > T and c.1219_1223 + 5del, is the patient with the highest value of homocysteine at diagnosis, partially responsive to vitamin B6 administration.

We are aware of the possibility of ENS false negatives in our center during the reference period due to an inappropriate Met cut-off, laboratory technical mistakes, or the presence of normal Met levels during the first days of life [14,15,16,17,18,19,20,21,22,23,24,25,26,27,28,29,30,31,32,33,34,35,36,37,38,39,40,41,42,43,44,45,46,47,48,49,50,51,52,53,54,55,56,57,58,59,60,61,62,63,64,65,66,67]. Nevertheless, today we cannot confirm any false negative ENS results because we have not carried out yet a regional survey to find clinically CBSD-diagnosed patients during the observation period.

Newborns with CBSD often undergo a pyridoxine trial with a high dose of pyridoxine to determine B_6_ responsiveness. This trial is not without side effects, as demonstrated by several case reports showing the onset of respiratory failure and rhabdomyolysis during a pyridoxine challenge [68]. These potentially lethal side effects highlight the need for carefully redacted protocols with a safe weight-based dosage of pyridoxine, indications for test duration, and clinical-laboratory monitoring during the testing period in infants. In our experience, we did not observe any side effects during the trial. All our patients required diet therapy and betaine to achieve safe values of tHcy < 100 μmol/L. This is not surprising because guidelines consider dietary management as the primary therapeutic approach for pyridoxine-unresponsive patients and an additional treatment, other than pyridoxine, for partially responsive patients [1]. Moreover, as reported by the European survey of Adam S. et al. and by 2017 guidelines the most common treatment for unresponsive patients is a combination of diet and betaine [1,54]. The 2017 guidelines recommend keeping the p-homocysteine concentration below 100 μmol/L [1]. More tight control may provide benefits but, at present, this is unproven. From our experience, it seems that patients who start with higher tHcy tend to maintain a higher trend over time than the other cases. These patients could benefit from a Met washout lasting for a variable number of days [69]. Certainly, it is mandatory to carefully evaluate plasma amino acid levels (i.e Met) during the follow-up to avoid damages related to the amino acid excess or deficiency [70,71]. We need internationally shared protocols for the management of hypermethioninemia, on how to carry out the clinical-instrumental follow-up, and on the impact of intensive care on quality of life. We need answers to crucial questions such as when to perform a magnetic resonance of the central nervous system to detect any early complications or how often to perform an extensive ophthalmological examination.

It is essential to remember that, despite the presence of the ENS, the classic clinical signs of this pathology (lens dislocation, thromboembolism, intellectual disability, psychiatric disorders, osteoporosis) must be well known by clinicians in any case. ENS has only been active for newborns for a few years and the problem of false negatives remains an open question still without a clear solution.

## Figures and Tables

**Table 1:** Laboratory values of the first DBS and diagnostic confirmation. Plasma HCY normal value 5–15 μmol/L. Plasma Met normal value 20–26 μmol/L. Met DBS normal value < 45 μmol/L if birth weight > 1800 g. DBS Hcy: positive > 5 μmol/L.

	Gestational Age Birth Weight	I DBS MET μmol/L	2TT DBS Hcy	Plasma tHcy (μmol/L)	Plasma MET (μmol/L)
Patient 1	38 weeks				
		102.51	Positive	95.6	727.15
	3840 g				
Patient 2	40 weeks 3895 g	45.02	Positive	105.8	93.46
Patient 3	39 weeks				599.99
	3030 g	79.12	Positive	204.3	

**Table 2:** Patient ethnic group and genotypes. HGMD: Human Gene Mutation Database. ACMG: American College of Medical Genetics and Genomics. P: Pathogenetic. LP: Likely Pathogenetic. A: Absent. US: Uncertain Significance. LB: Likely Benign. B: Benign.

	Ethnic Group	Variant Allele V1	Variant Allele V2	HGMD	ACMG	Reference
Patient 1	North Africa					
		c.969 G > A	c.969 G > A	P	P	[60]
Patient 2	Caucasian	c.770 C > T	c.1330 G > A	V1: P V2: P	V1: LP V2: LP	V1: [61] V2: [62]
Patient 3	Caucasian					
				A	V1: LP V2: LP	A
		c.1333 C > T	c.1219_1223 + 5del			

**Table 3:** Response to Pyridoxine trial, current therapy e metabolic control in our cases. PR = Partial Responder, ff the tHcy falls > 20% but remains above 50 μmol/L. NR = Non-Responder, if the tHcy falls < 20% on pyridoxin (plasma homocysteine normal value 5–15 μmol/L).

	Plasma tHcy after 14 days Pyridoxine Trial μmol/L	Current Treatment
Patient 1	83.5 (−12.6%, NR)	Pyridoxine (10 mg/kg/day) + OH-B12(1000 mcg) + Folate (5 mg) from the onset. Diet therapy from 1 month of life (Met 9 mg/kg, Natural Protein Intake 0.57 g/kg/die, Total Protein Intake 2.2 g/kg/die). Betaine (80 mg/kg) from 45 days of life
Patient 2	76.9 (−27%, PR)	Pyridoxine (10 mg/kg/day) + OH-B12(1000 mcg) + Folate (5 mg) from the onset. Diet therapy from 2 months of life (Met 20 mg/kg, Natural Protein Intake 1.2 g/kg/die, Total Protein Intake 2.6 g/kg/die). Betaine (90 mg/kg) from 75 days of life
Patient 3	113.3 (−44%, PR)	Pyridoxine (10 mg/kg/day) + OH-B12 (1000 mcg) + Folate (5 mg) from the onset. Diet therapy from 1 month of life (Met 10 mg/kg, Natural Protein Intake 0.7 g/kg/die, Total Protein Intake 2.7 g/kg/die). Betaine (105 mg/kg) from 60 days of life

## Data Availability

All clinical data and materials are available in our Pediatric Unit.
